# Translating Molecular Insights into Effective Targeting of Glioblastoma Stem Cells

**DOI:** 10.3390/cancers18050860

**Published:** 2026-03-07

**Authors:** Shilpi Singh, Deepak Singh Kapkoti, Gatikrushna Singh

**Affiliations:** 1 Department of Neurosurgery, University of Minnesota, Minneapolis, MN 55455, USA; 2 CSIR-Central Institute of Medicinal & Aromatic Plants, Lucknow 226015, India; 3 SBRB Inter College, Pali 241123, India

**Keywords:** glioblastoma stem cells, tumor microenvironment, therapeutic resistance, non-coding RNA

## Abstract

Glioblastoma stem cells (GSCs) function as dynamic regulators of tumor persistence, maintained by interconnected genetic, epigenetic, metabolic, and microenvironment-derived circuits. Rather than fixed entities, GSCs continuously recalibrate their functional state as transcriptional regulators, chromatin architecture, and non-coding RNA networks shift in response to microenvironmental cues. Hypoxic, vascular, and immune niches reinforce these adaptive states by stabilizing HIF signaling, modulating cytokine gradients, and sustaining immunosuppression. Metabolic flexibility further supports survival under therapeutic and environmental stress. Standard therapies inadvertently activate these same resilience pathways: TMZ enhances DNA repair and quiescent survival, while radiation promotes mesenchymal transition and immune evasion, thereby enriching GSC-associated circuits that drive recurrence. Understanding how these molecular circuits converge to sustain stemness, plasticity, and microenvironmental crosstalk highlights the need for combinatorial strategies that simultaneously disrupt epigenetic gating, metabolic rewiring, ncRNA-controlled repair, and niche-dependent signaling to achieve durable glioblastoma control.

## Simple Summary

Glioblastoma stem cells (GSCs) play a central role in glioblastoma initiation, heterogeneity, therapeutic resistance, and recurrence. Rather than representing a fixed population, GSCs exhibit dynamic and reversible cellular states shaped by genetic and epigenetic programs and by signals from hypoxic, vascular, and immune niches. Advances in single-cell and spatial profiling have revealed extensive GSC plasticity, including transitions between proliferative and quiescent states and between proneural and mesenchymal phenotypes. These adaptive programs are reinforced by metabolic flexibility, immune suppression, and regulatory networks controlled by non-coding RNAs. Importantly, standard therapies can inadvertently promote GSC-associated survival mechanisms, contributing to treatment failure. This review integrates current insights into the molecular and microenvironmental circuits that sustain GSC identity and resilience, and discusses emerging therapeutic strategies aimed at disrupting these networks through combinatorial targeting of stemness, metabolism, epigenetic regulation, and immune evasion to enable more durable glioblastoma control.

## 1. Introduction

Cancer is no longer considered a uniform expansion of proliferating cells but as an ecosystem composed of distinct, interacting neoplastic populations. Among these, a stem-like subpopulation known as cancer stem cells (CSCs) represents a functionally dominant category that drives tumor initiation, sustains heterogeneity, promotes invasion and metastasis, and mediates resistance to therapy [1,2,3]. In the central nervous system, glioblastoma, a grade IV diffuse glioma, stands as the most compelling example of this biology. Despite being relatively rare, GBM accounts for nearly half of all malignant CNS tumors and displays highly aggressive clinical behavior, poor therapeutic response, and a median overall survival of approximately 12–15 months [4,5,6,7]. Traditionally, GBM research focused on bulk tumor tissues, often overlooking extensive intratumoral diversity that underlies treatment failure. The identification of glioblastoma stem cells (GSCs) over the past two decades fundamentally reshaped this perspective. GSCs possess self-renewal capacity, multilineage differentiation potential, and the ability to regenerate tumors that recapitulate patient disease [8,9]. Their distinct genetic, epigenetic, and transcriptional programs, including dysregulated Notch, Hedgehog, and Wnt signaling, support a stem-like state that enables therapeutic resistance and tumor recurrence [10,11].

Advances in single-cell and spatial profiling have further revealed that GSCs comprise multiple molecularly defined subtypes with unique biological behaviors [12,13]. These insights have catalyzed precision medicine strategies aimed at dismantling GSC maintenance networks, exploiting lineage-specific vulnerabilities, and integrating immunotherapeutic approaches. Targeting the molecular and functional diversity of GSCs is thus critical for improving GBM management, offering the potential to curb recurrence, enhance therapeutic efficacy, and advance personalized treatment paradigms in this highly aggressive disease [14,15,16].

This review synthesizes mechanistic advances illustrating how GSC-intrinsic regulatory networks and niche-derived signals converge to maintain stemness, drive phenotypic transitions, and reinforce therapy resistance in GBM. By integrating emerging insights across developmental signaling pathways, epigenetic regulation, metabolic adaptation, and immune modulation, we delineate key vulnerabilities that can be leveraged through rational combinatorial strategies. A deeper understanding of GSC plasticity and microenvironmental integration will be essential for designing durable, precision-based interventions capable of altering the clinical trajectory of this highly lethal tumor.

## 2. A Comprehensive Insight into Glioblastoma Stem Cell Biology

CSCs were first identified in acute myeloid leukemia by Bonnet and Dick in 1997 as rare, self-renewing cells capable of initiating tumors. In glioblastoma, analogous GSCs were subsequently validated through their ability to regenerate tumors in xenograft models. GSCs are enriched using markers such as CD133, CD15, CD56, SRY-box transcription factor 2 (SOX2), and SOX9 (Table 1) and are distinguished from stromal populations by the absence of CD248, CD105, and alpha-smooth muscle actin (αSMA) [17,18]. Gene-expression signatures [19,20,21], metabolic features [22,23], and biological properties [24] reveal interconnected GSC subtypes. Functionally, GSCs span a proliferative-quiescent axis: proliferative GSCs (pGSCs) drive rapid tumor growth, whereas quiescent GSCs (qGSCs) remain dormant, exhibit marked resistance to radiotherapy and chemotherapy, and act as a reservoir for recurrence. Therapeutic stress can trigger interconversion between these states, highlighting pronounced cellular plasticity [25,26,27,28].

Transcriptomic and metabolomic profiling further stratifies GSCs into proneural (PN) and mesenchymal (MES) subtypes. PN GSCs are highly proliferative, predominantly glycolytic, and often harbor isocitrate dehydrogenase 1 (IDH1) mutations. They reside in perivascular niches and express markers such as CD133, CD15, DLL3, MAP2, SOX2, oligodendrocyte transcription factor 2 (OLIG2), and enhancer of zeste homolog 2 (EZH2). In contrast, MES GSCs display metabolic flexibility, transitioning between glycolysis and oxidative phosphorylation, and show enhanced invasiveness and radio-chemoresistance, partly through glutamine dependence. MES GSCs, typically IDH1-wildtype, localize to hypoxic and necrotic regions and express CD44, aldehyde dehydrogenase 1 family member A3 (ALDH1A3), epidermal growth factor receptor (EGFR), chitinase-3-like protein 1 (YKL40), B lymphoma Mo-MLV insertion region 1 (BMI1), and glial fibrillary acidic protein (GFAP) [29,30,31]. Temozolomide (TMZ) exposure can drive PN-to-MES transition, representing a major adaptive resistance mechanism.

Distinct RNA-splicing patterns and lncRNA programs further regulate pathways controlling cell-cycle progression, DNA repair, and ciliogenesis [32,33,34,35]. GSC-associated markers contribute directly to stemness, invasion, and therapy resistance [36,37,38]. While CD133-positive cells demonstrate robust sphere formation, tumorigenicity, and drug resistance, CD133 expression is dynamic, and CD133-negative cells can reacquire stem-like properties through epigenetic reprogramming [39,40,41]. Single-cell analyses have also identified CD133-independent GSC populations driven by pathways such as Notch3 [42,43].

Core transcription factors, including octamer-binding transcription factor 4 (OCT4), SOX2, and NANOG, cooperatively sustain self-renewal, proliferation, migration, and immune evasion [44,45,46,47]. CD44 is associated with invasiveness, hypoxia-induced plasticity, and radiochemoresistance [48,49]. CD109, enriched in perivascular GSCs, interacts with glycoprotein 130 (GP130) to activate interleukin-6 (IL-6)/signal transducer and activator of transcription 3 (STAT3) signaling, promoting stemness and chemoresistance. Integrin α2 correlates with STAT3 activation and induction of EMT-like programs [50,51,52,53,54,55]. Cellular prion protein (PrPC), upregulated in neurosphere cultures and co-localized with CD133, is essential for maintaining stem cell markers, adhesion molecules, migration, and tumor growth; its interaction with Hsp70/Hsp90 organizing protein (HOP) further enhances GSC proliferation and self-renewal [56].

**Table 1:** GSC markers and their role in resistance.

Marker	Functional Category	Mechanism of Resistance	Primary Therapy Affected	References
CD133 (PROM1)	Stemness/tumor initiation	Enhanced DNA damage checkpoint activation, Enrichment of radioresistant tumor-initiating cells	Radiation, TMZ	[57,58]
CD44	Mesenchymal transition/adhesion	Activation of STAT3 and PI3K-AKT signaling. Promotes invasion and survival	Radiation, TMZ	[59,60]
CD15	Stem/progenitor marker	Marks tumor-propagating, therapy-resistant populations. Associated with enhanced clonogenicity	Radiation, Chemotherapy	[61,62]
CD90 (THY1)	Mesenchymal/stemness-associated glycoprotein	Associated with invasive phenotype and treatment resistance via integrin and TGF-β signaling	Chemotherapy	[63,64]
ITGA6	Integrin/niche interaction	Promotes adhesion to vascular niche, Enhances survival signaling and self-renewal	Radiation	[65]
ALDH1A1	Metabolic detoxification	ROS scavenging, Protection from alkylating-agent-induced oxidative stress	Chemotherapy	[66,67]
SOX2	Pluripotency transcription factor	Maintenance of self-renewal, Resistance to genotoxic stress	Radiation, TMZ	[68,69,70]
OLIG2	Lineage transcription factor	Suppression of p53-mediated apoptosis. Enhanced DNA repair signaling	Radiation	[71,72]
Nestin (NES)	Neural progenitor marker	Correlates with proliferative, Therapy-resistant phenotype	Radiation	[73,]
L1CAM (CD171)	Adhesion/survival signaling	Increased DNA repair capacities Promotes radioresistance	Radiation	[74]
BMI1	Epigenetic regulator (Polycomb)	Chromatin remodeling, Maintenance of stemness. Enhanced DNA repair	Radiation	[75,76]

GSC distribution is tightly shaped by interactions with the surrounding tumor microenvironment. Higher-grade gliomas exhibit increased GSC abundance, particularly within perivascular niches that mirror neural stem cell compartments [8,77,78]. Endothelial cells and mesenchymal stem cells supply paracrine cues such as endothelin-1-mediated AKT activation, which regulate GSC self-renewal and differentiation, while vascular networks provide essential nutrients and oxygen [79,80]. Hypoxic regions further activate HIF-1α-dependent programs that drive angiogenesis, metabolic reprogramming, Wnt/β-catenin signaling, and the upregulation of CD133 and VEGF, collectively enhancing neurosphere formation, invasion, and treatment resistance [81,82,83]. Inflammatory cytokines and chemokines released by macrophages and oligodendrocyte progenitor cells at the invasive margin additionally promote GSC adhesion, migration, and niche remodeling [84]. Together, intrinsic stemness programs and microenvironment-derived signals integrate to sustain GSC plasticity, preserve intratumoral heterogeneity, and reinforce therapeutic resistance in GBM.

## 3. How Stem Cell Networks Fuel Tumor Growth

GSCs are sustained by integrated oncogenic signals, genetic and epigenetic alterations, metabolic adaptability, and continuous crosstalk with the tumor microenvironment (TME). Together, these mechanisms reinforce self-renewal, therapy resistance, and aggressive tumor progression.

### 3.1. Intrinsic Regulatory Programs Governing GSC Identity and Plasticity

Key developmental programs, including Sonic Hedgehog (SHH), Notch, and Wnt, are aberrantly reactivated in GSCs. The SHH pathway, essential during embryogenesis but largely silent in adult tissues, becomes re-engaged to promote self-renewal, proliferation, and chemoresistance. SHH-GLI signaling increases the expression of drug efflux pumps (ABCB1, ABCG2, ABCC1) and DNA repair proteins such as O6-methylguanine-DNA methyltransferase (MGMT) [85,86,87,88,89,90], while directly inducing NANOG and suppressing bone morphogenetic protein (BMP) signaling to block differentiation. Loss of TP53 further amplifies SHH activity. Importantly, SHH inhibition sensitizes GSCs to TMZ, underscoring its therapeutic relevance [91].

Notch signaling, a critical regulator of neural stem cell fate, is similarly hyperactivated in GSCs. Nuclear translocation of the Notch intracellular domain (NICD) induces HES and HEY transcription factors that maintain self-renewal [92]. Tumor microenvironment-derived factors such as Tenascin-C further enhance Notch activity. CD133^+^ GSCs display elevated expression of Notch-associated activators, including ID4 and FABP7, which promote invasion and tumor aggressiveness [93,94,95,96]. The Wnt pathway additionally reinforces stemness and tumor propagation [97]. Transcription factors such as PLAG1-like zinc finger 2 (PLAGL2) and forkhead Box M1 (FOXM1) increase Wnt signaling and expression of stemness-associated genes, while epigenetic regulators, including EVI, modulate canonical and noncanonical cascades [98,99,100]. Endothelial differentiation induced by Wnt5A enhances neovascularization and supports tumor expansion [101]. Collectively, these developmental programs maintain stem-like phenotypes and continuous tumor growth.

Genetic and epigenetic dysregulation provides additional layers of plasticity. Reduced histone H3 lysine 9 trimethylation (H3K9me3) levels and altered activity of chromatin modifiers such as lysine-specific demethylase 4A (KDM4A), EZH2, and DNA methyltransferase 3 alpha (DNMT3A) sustain transcriptional flexibility [102,103,104,105]. TERT promoter mutations enable telomere extension and cellular immortalization [106]. EGFRvIII activates persistent phosphatidylinositol 3-kinases (PI3K)/protein kinase B (AKT) signaling, promoting proliferation, survival, and metabolic rewiring, especially under hypoxic conditions where hypoxia-inducible factor 1-alpha (HIF-1α) cooperates to upregulate enzymes such as pyruvate kinase M2 (PKM2) and lactate dehydrogenase A (LDHA) [107,108]. Loss of alpha-thalassemia X-linked (ATRX) intellectual disability protein disrupts chromatin remodeling and enhances hypoxic survival [109,110], whereas TP53 mutations impair DNA damage response and increase genomic instability. IDH1 mutations, particularly R132H, produce the oncometabolite 2-hydroxyglutarate (2-HG), which alters redox homeostasis, remodels the epigenome, and blocks differentiation, thereby enabling gliomagenesis [111].

### 3.2. Microenvironmental and Metabolic Adaptation Supporting GSC Persistence

GSCs also integrate immunomodulatory signals to promote tumor persistence. Tumor-associated macrophages (TAMs), particularly M2-like subsets, reinforce GSC maintenance via paracrine factors. TAM-derived pleiotrophin (PTN) activates GSCs through protein tyrosine phosphatase receptor type Z1 (PTPRZ1), with PTN levels correlating with macrophage infiltration and poor prognosis [112]. TGF-β1 secreted by TAMs enhances GSC invasiveness through transforming growth factor beta receptor 2 (TGFBR2)-dependent induction of matrix metalloproteinase-9 (MMP-9) [113]. In parallel, GSCs evade immune surveillance by downregulating NK-cell-activating ligands, increasing inhibitory signals, and recruiting immunosuppressive populations. By remodeling chemokine gradients, particularly through the C-X-C motif chemokine ligand 12 (CXCL12)-C-X-C motif chemokine receptor 4 (CXCR4) axis, GSCs migrate to perivascular niches and maintain stemness [114]. Immune-rich tumor regions intensify transcriptional heterogeneity and may contribute to resistance to immune checkpoint blockade.

Metabolic plasticity is a hallmark of GSCs, enabling dynamic switching among glycolysis, oxidative phosphorylation, and the pentose phosphate pathway (PPP). Under hypoxia, glycolysis predominates, while the PPP provides NADPH to maintain redox balance and support biosynthesis [115]. Transaldolase links PPP metabolism to oxidative stress regulation and tumor progression. During glucose scarcity, GSCs upregulate high-affinity glucose transporters (GLUT family) to secure nutrient uptake [116,117]. Beyond glucose metabolism, fatty acid oxidation and glutamine metabolism supply energy and promote an immune-permissive microenvironment. IDH1 mutation-driven 2-HG production further reprograms cellular metabolism and epigenetic regulation, reinforcing stemness and tumor persistence [118,119]. The perivascular niche provides essential support for GSC survival, proliferation, and therapeutic resistance. Endothelial cells release soluble factors that reinforce stemness and radiochemoresistance [120]. Direct GSC-endothelial contact induces phosphodiesterase 7B (PDE7B), expanding stem-like populations and correlating with poor patient survival [121]. GSC-derived extracellular vesicles and exosomes modulate immune responses by reprogramming immune cells and enhancing immune evasion [122,123]. Pericytes—often derived from GSCs—contribute to vascular stability and neovascularization, thereby sustaining tumor growth [124,125].

## 4. Role of GSCs in Treatment Failure

**Figure 1:**
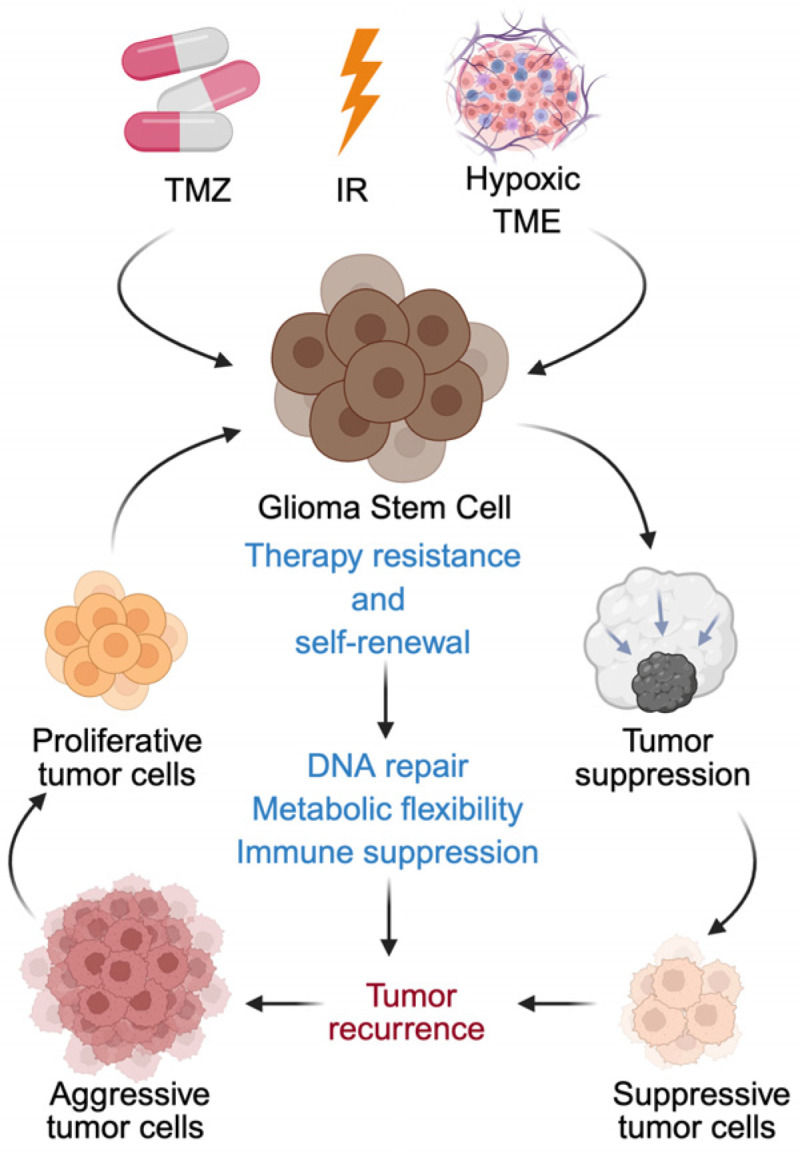
Glioma stem cells drive therapeutic resistance and tumor recurrence in glioblastoma. Standard therapies, including temozolomide (TMZ) and radiation, together with the hypoxic tumor microenvironment (TME), impose selective pressure on glioblastoma cells. While bulk differentiated tumor cells are largely eliminated, glioma stem cells (GSCs) persist due to intrinsic resistance mechanisms, including enhanced DNA repair capacity, metabolic flexibility, and immunosuppressive signaling. Therapeutic stress and microenvironmental cues further promote phenotypic plasticity and dedifferentiation of non-stem tumor cells into a stem-like state, reinforcing the GSC pool. Surviving GSCs sustain minimal residual disease and repopulate the tumor, ultimately driving recurrence and progression to a more aggressive GBM phenotype. This schematic highlights GSCs as central mediators of treatment failure and key targets for durable therapeutic intervention.

GSCs constitute a highly adaptable and therapy-resistant subpopulation that fundamentally drives GBM progression, recurrence, and treatment failure. In contrast to differentiated tumor cells, GSCs possess superior DNA repair capacity, metabolic flexibility, and robust immunomodulatory functions, allowing them to withstand chemoradiotherapy and repopulate the tumor (Figure 1). Increasing evidence indicates that the hostile TME and exposure to standard treatments, including TMZ and radiation, can further enhance GSC phenotypes or induce dedifferentiation of non-stem glioma cells, amplifying therapeutic resistance [8,28,126,127,128,129]. These multidimensional defense mechanisms establish GSCs as central architects of GBM aggressiveness and underscore the need to decipher their survival strategies to develop durable therapeutic interventions.

### 4.1. Intrinsic Resistance Mechanisms

GSCs rely on a constellation of intrinsic mechanisms that confer potent resistance to therapy. Enhanced DNA repair is a major contributor. MGMT, a key mediator of TMZ resistance, shows variable expression across GBM but is generally associated with increased resistance, including in CD133^+^ GSCs [130,131,132,133,134,135,136,137]. However, MGMT-independent mechanisms, particularly the upregulation of ATP-binding cassette (ABC) transporters, provide an additional layer of chemoresistance. Elevated multidrug resistance 1 (MDR1), ABCG2/BCRP, and ABCB1 expression in GSCs enables drug efflux and reduces intracellular TMZ accumulation [138,139,140,141]. Pharmacological inhibition of ABC transporters or epigenetic suppression of their promoters sensitizes GSCs to TMZ and other chemotherapeutics [142,143,144,145].

**Figure 2:**
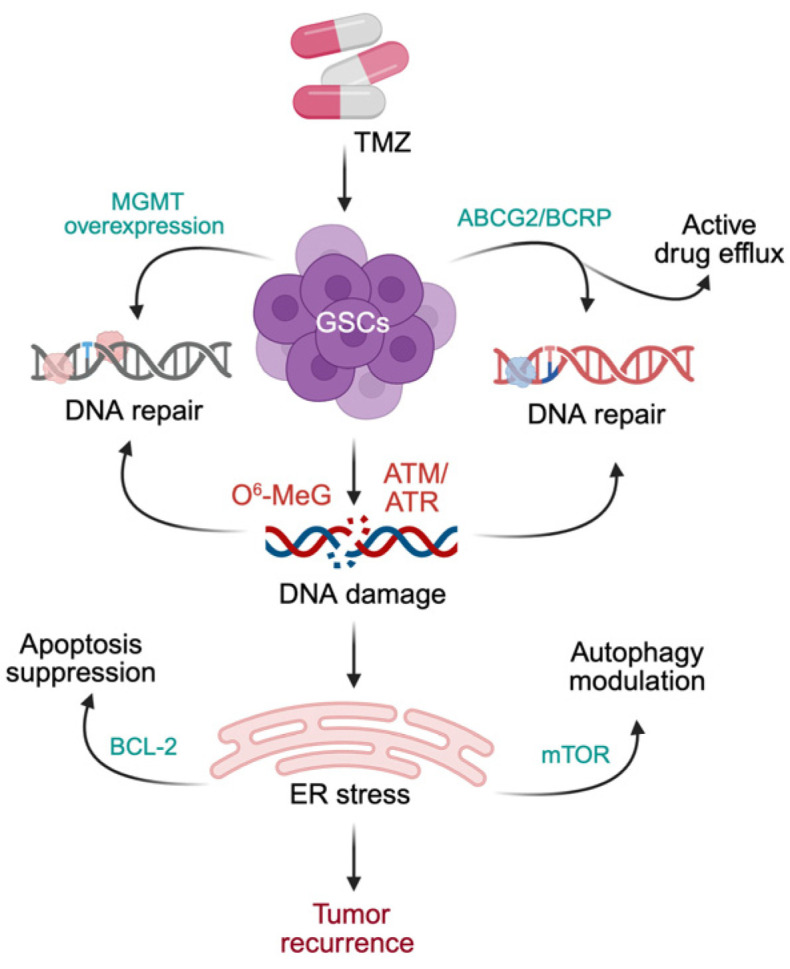
Intrinsic molecular mechanisms driving therapy resistance in glioma stem cells. Glioma stem cells (GSCs) exhibit multiple intrinsic resistance pathways that enable survival following temozolomide (TMZ) and radiation therapy. Enhanced DNA damage repair, mediated by MGMT-dependent O^6^-methylguanine repair and activation of homologous recombination pathways and checkpoint signaling, allows efficient resolution of therapy-induced DNA lesions. Concurrently, overexpression of ABC transporters promotes drug efflux and reduces intracellular TMZ accumulation. GSCs also engage anti-apoptotic signaling pathways, including Bcl-2 upregulation, and exhibit dynamic modulation of autophagy through mTOR signaling, contributing to stress adaptation. Together, these coordinated mechanisms sustain a therapy-resistant GSC pool that persists after treatment and drives tumor recurrence.

Resistance to apoptosis further strengthens GSC survival. Following TMZ exposure, GSCs induce anti-apoptotic pathways while suppressing caspase activation, collectively limiting treatment-induced cytotoxicity [146,147,148]. Autophagy contributes to therapy resistance in a highly context-dependent manner: some GSCs exhibit impaired canonical autophagy after TMZ exposure [149,150,151], whereas induced autophagy promotes differentiation, modulates stemness markers, and enhances therapy sensitivity in other models [152]. Radiation resistance follows similar patterns. CD133^+^ GSCs often demonstrate increased phosphorylation of checkpoint kinase 1 (CHK1)/CHK2/H2A histone family member X (H2AX) and upregulation of DNA repair genes including RAD51, breast cancer gene 1 (BRCA1), and BRCA2, facilitating efficient repair of radiation-induced damage [153,154,155,156,157]. However, radiosensitivity varies across models, suggesting contributions from additional extrinsic factors [158,159]. Collectively, these intrinsic mechanisms enhanced DNA repair, drug efflux, impaired apoptosis, and context-dependent autophagy create a robust survival platform enabling GSCs to endure and recover following cytotoxic therapy (Figure 2).

### 4.2. Microenvironment and Immune-Mediated Protection

**Figure 3:**
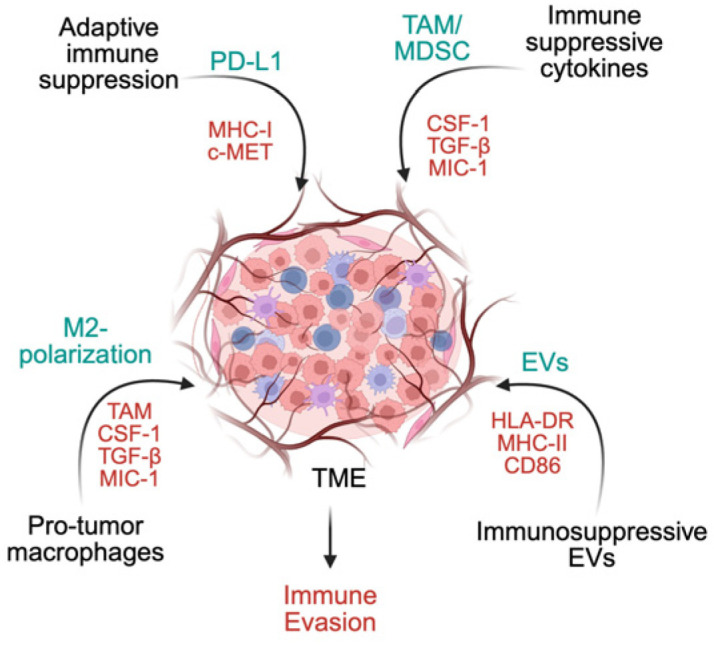
Microenvironmental and immune-mediated protection of glioma stem cells. Glioma stem cells (GSCs) evade immune surveillance and shape an immunosuppressive tumor microenvironment to sustain tumor growth and therapeutic resistance. GSCs suppress adaptive immunity through reduced antigen presentation (low MHC I, absent MHC II, CD40, CD80, CD86) and expression of inhibitory molecules such as PD-L1, which mediate contact-dependent T cell inhibition. They secrete immunosuppressive cytokines, including CSF-1, TGF-β, MIC-1, and galectin-3, that promote recruitment and polarization of tumor-associated macrophages (TAMs) and myeloid-derived suppressor cells (MDSCs) toward tumor-supportive M2-like phenotypes. GSC-derived extracellular vesicles (EVs), enriched in PD-L1, further suppress T cell activation and reprogram monocytes, amplifying systemic immune suppression. Collectively, these mechanisms create an immune-privileged niche that protects GSCs, facilitates immune evasion, and promotes tumor progression and recurrence.

GSCs actively sculpt an immunosuppressive microenvironment and exploit niche-derived cues to enhance survival and undermine therapeutic response (Figure 3). They impair T cell activation by reducing antigen presentation, expressing inhibitory ligands (e.g., PD-L1), and secreting immunosuppressive mediators such as galectin-3, transforming growth factor-beta (TGF-β), and prostaglandin E2 (PGE2), collectively curtailing cytotoxic T cell function [160,161,162,163,164,165,166]. Immune suppression correlates more strongly with in vivo tumorigenic potential than with classical surface markers such as CD133 [167,168].

GSCs also reprogram innate immune populations. They recruit and polarize myeloid-derived suppressor cells (MDSCs) and TAMs toward tumor-supportive, M2-like states through the secretion of colony-stimulating factor 1 (CSF-1), TGF-β1, macrophage inhibitory cytokine-1 (MIC-1), and periostin (POSTN). POSTN-mediated TAM recruitment enhances invasion, tumor growth, and poor clinical outcomes [169,170,171,172,173]. GSC-conditioned media suppresses macrophage and microglial phagocytosis while increasing IL-10 and TGF-β1 in a STAT3-dependent manner, reinforcing local immune paralysis. Chemokines (CCL2, CXCL1, CXCL8, CCL5) shape immune cell trafficking, promoting accumulation of immunosuppressive cells and limiting NK- and T cell-mediated tumor clearance [174,175,176].

Extracellular vesicles (EVs) derived from GSCs amplify systemic immune suppression. EV-associated PD-L1 directly inhibits T cell activation and proliferation, an effect reversible by PD-1 blockade [177,178]. High PD-L1 DNA in circulating EVs correlates with tumor burden, highlighting its utility as a biomarker [179]. Mesenchymal niches enriched in PD-L1 and c-MET further intensify immune evasion.

The specialized TME also modulates therapeutic response. Hypoxia, stromal interactions, metabolic gradients, and cytokine signaling collectively enhance radioresistance, restrict T cell infiltration, and impose metabolic constraints such as glucose depletion and accumulation of immunosuppressive metabolites (adenosine) that impair antitumor immunity [153,180,181,182]. Through these processes, GSCs establish a multilayered immune-evasive network that sustains stemness, supports tumor progression, and diminishes therapeutic efficacy.

### 4.3. Adaptive Plasticity and Phenotypic Transition

**Figure 4:**
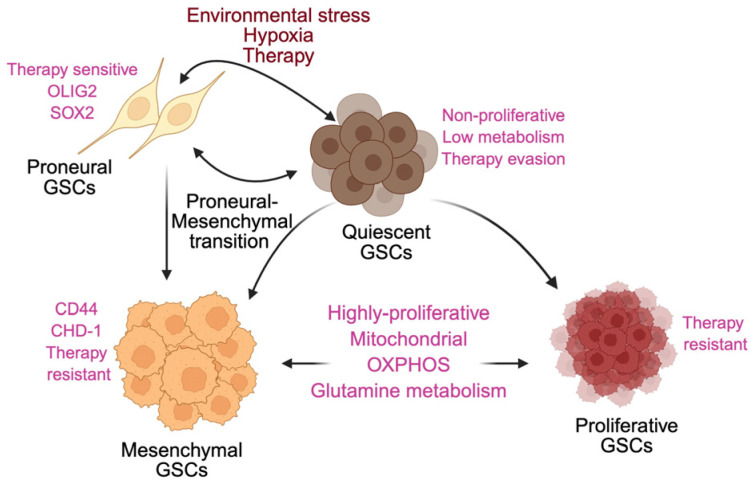
Adaptive plasticity and phenotypic transitions in glioma stem cells. Glioma stem cells (GSCs) display dynamic phenotypic plasticity that contributes to tumor persistence and therapy resistance. Under environmental stressors such as hypoxia, nutrient deprivation, or treatment exposure, proliferative GSCs (pGSCs) can transition into a quiescent state (qGSCs), characterized by reduced proliferation, low metabolic activity, and enhanced stress tolerance. Quiescent GSCs serve as a reservoir for tumor recurrence and can re-enter the cell cycle through activation of cell-cycle regulators. In parallel, radiotherapy can induce a proneural-to-mesenchymal transition, generating mesenchymal GSCs with elevated expression of resistance-associated genes and increased reliance on mitochondrial oxidative phosphorylation and glutamine metabolism. These bidirectional transitions enable GSCs to evade cytotoxic stress, regenerate tumor populations, and drive recurrent, therapy-resistant glioblastoma.

A defining hallmark of GSCs is their remarkable plasticity, enabling dynamic transitions between cellular states that circumvent therapy (Figure 4). One adaptive axis involves reversible switching between proliferative (pGSC) and quiescent (qGSC) states. Under hypoxia, nutrient deprivation, or therapy-induced stress, pGSCs enter quiescence, reducing metabolic activity and evading cytotoxic injury. These qGSCs retain the ability to re-enter the cell cycle when conditions improve, repopulating the tumor and sustaining recurrence. The GINS complex (SLD5, PSF1-3) coordinates cell-cycle reactivation and drives re-entry into proliferation [22,35,183,184,185].

A second major adaptive transition is the proneural-to-mesenchymal (PN → MES) shift, strongly induced by radiotherapy. PN GSCs exposed to radiation acquire mesenchymal traits associated with enhanced resistance, invasiveness, and metabolic reprogramming toward glutamine utilization and oxidative phosphorylation (OXPHOS) dependence [27,29,136,186,187,188,189]. Targeting pathways that mediate this transition restores radiosensitivity in preclinical models and represents an emerging therapeutic avenue. Together, these adaptive programs quiescence, PN → MES transition, and metabolic reconfiguration allow GSCs to survive therapeutic insult, regenerate the tumor, and maintain long-term resistance.

## 5. Integrated Regulatory Networks of Non-Coding (ncRNAs) RNAs in GSCs

Mounting evidence demonstrates that microRNAs (miRNAs), long non-coding RNAs (lncRNAs), and circular RNAs (circRNAs) form highly interconnected regulatory networks that converge on the core signaling pathways sustaining GSCs (Table 2). Rather than acting in isolation, these RNA species coordinate transcriptional, epigenetic, metabolic, and post-transcriptional programs that preserve stemness, enhance invasion, and drive therapeutic resistance in glioblastoma. A unifying feature of these networks is their convergence on key developmental cascades, including Notch, Wnt/β-catenin, Hedgehog, and EGFR-STAT3-positioning ncRNAs as master regulators of GSC identity.

Tumor-suppressive miRNAs such as miR-137, miR-124, miR-128, and miR-326 restrict GSC maintenance by targeting central stemness regulators. miR-137 inhibits the testis-specific, vespid, and pathogenesis protein 1 (RTVP-1)/CXCR4 axis to suppress self-renewal and invasion, while miR-128 family members downregulate BMI1 and E2F3, limiting proliferation. miR-326 represses smoothened, frizzled class receptor (SMO), attenuates Hedgehog signaling, and reduces tumorigenicity [186,187,188]. In contrast, oncogenic lncRNAs and circRNAs reinforce these same pathways. INHEG enhances translation of EGFR, insulin-like growth factor 1 receptor (IGF1R), and PDGFRB [189]; FOXD2-AS1 activates Notch signaling through TAF-1 recruitment [190]; and LINC01503 stabilizes GLI2 to potentiate Wnt/β-catenin signaling [191]. MALAT1 sustains SOX2 expression while integrating multiple developmental pathways [192,193]. Among circRNAs, circ-E-Cad encodes an oncogenic E-cadherin variant that activates EGFR–STAT3 signaling independent of ligand stimulation [194], whereas circASPM promotes proliferation via the miR-130b-3p/E2F1 axis [195]. Together, these multilayered interactions construct a robust RNA-centered architecture that stabilizes stem-like identity and developmental plasticity.

Non-coding RNAs also integrate microenvironmental cues to regulate the GSC niche. LUCAT1 forms a transcriptional complex with HIF1α and CBP to amplify hypoxia-responsive genes and maintain stemness under low-oxygen conditions [196]. circARF1 enhances angiogenesis through a miR-342-3p/ISL2/VEGFA circuit, establishing a feedback loop that sustains ERK signaling [197]. miR-148a and miR-31 modulate factor-inhibiting hypoxia-inducible factor 1 (FIH1), HIF1α, and Notch pathway components to regulate vascular niche dynamics [198]. Additional ncRNAs modulate extracellular matrix remodeling and mesenchymal transition; for example, miR-20a and miR-106a enhance invasiveness by suppressing tissue inhibitor of metalloproteinases-2 (TIMP-2), while H19 and CRNDE promote MES-like traits through miRNA sponging and activation of pro-invasive transcriptional programs [199]. circKPNB1 reinforces nuclear factor kappa-light-chain-enhancer of activated B cells (NF-κB) signaling via a SPI1/TNF-α loop, strengthening inflammatory and invasive phenotypes [200]. These diverse circuits underscore ncRNAs as central mediators linking hypoxia, inflammation, metabolic adaptation, and invasiveness.

Non-coding RNAs are further embedded within feedback networks that buffer GSCs against chemotherapy and radiotherapy. TP73-AS1 enhances TMZ resistance by regulating ALDH1A1 and metabolic pathways [201]. MALAT1 and NEAT1 promote DNA repair, apoptosis resistance, and stemness programs [202]. LincRNA-p21 modulates β-catenin signaling and radiosensitivity through the miR-146b-5p/HuR axis [203]. circRPPH1 and circKPNB1 stabilize stemness-associated transcription factors and maintain oncogenic signaling [204]. These interconnected systems generate resilient, self-amplifying regulatory loops that ensure GSC survival under therapeutic stress.

A central mechanistic theme linking these interactions is the competing endogenous RNA (ceRNA) model, wherein lncRNAs and circRNAs sequester tumor-suppressive miRNAs, thereby derepressing oncogenic targets. Examples include SOX2OT regulation of SOX2 via miR-194-5p/miR-122, HOTAIR-mediated repression of PDCD4 through EZH2/LSD1 recruitment, and numerous circRNAs that maintain E2F1, GLI family zinc finger 2 (GLI2), and Spi-1 proto-oncogene (SPI1) by acting as miRNA sponges. Positive feedback loops further amplify these ceRNA interactions, strengthening oncogenic outputs and consolidating GSC identity.

The convergence of ncRNAs on EGFR-STAT3, Wnt/β-catenin, Notch, Hedgehog, NF-κB, and HIF1α signaling highlights their potential as actionable therapeutic nodes. Restoring tumor-suppressive miRNAs through mimics or epigenetic reactivation could simultaneously downregulate multiple stemness pathways. Conversely, antisense oligonucleotides, RNA interference, or CRISPR-based approaches targeting oncogenic lncRNAs and circRNAs may effectively disrupt core regulatory hubs. Importantly, the association of many ncRNAs with patient prognosis and treatment response supports their utility as biomarkers for patient stratification, therapeutic monitoring, and precision oncology in glioblastoma.

**Table 2:** Non-coding RNAs involved in GSCs modulation.

Non-Coding RNAs	Expression	Target	Mechanisms	References
miR-137	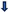	RTVP-1	Promotes neural differentiation and reduces GSC self-renewal suppressing stemness markers	[186]
miR-128	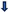	BMI1/E2F3	Limiting GSC stemness	[187,205]
miR-326	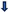		Suppresses GSC self-renewal, promotes partial differentiation, and decreases intracranial tumorigenicity	[188]
miR-20a miR-106a	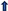	TIMP-2	Reduces GSC invasiveness	[199]
miR-148a miR-31	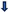	FIH1, HIF1α, and Notch	Sustaining stem cell populations Supporting aberrant vascular niches	[198]
miR-152	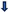	KLF4	Inhibits GSC proliferation, migration, and invasion, and promotes apoptosis	[206]
miR-124 miR-137	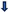		Triggers neuronal-like differentiation Induces G1 arrest reducing CDK6 and phosphorylated Rb levels	[207]
miR-29	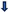	PDGFA	Inhibit GSC growth, invasion, and migration	[208]
miR-93	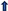	BECN1/Beclin 1, ATG5, ATG4B, and SQSTM1/p62	Regulate autophagy	[209]
miR-451	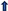	SMAD	Inhibit neurosphere formation, reduce tumorigenicity	[210]
miR-145	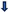	OCT4/SOX-2	Increase sensitivity to radiation and Temozolomide	[211]
INHEG	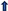		Promotes self-renewal Enhanced protein translation	
LincRNA-p21	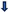	β-catenin	Enhanced β-catenin activity, stemness, and radioresistance	[203]
LUCAT1	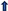		Promoting GSC adaptation and self-renewal	[196]
TP73-AS1	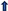	ALDH1A1	Enhanced TMZ resistance	[201]
FOXD2-AS1	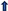	NOTCH	Promotes GSC stemness and proliferation, inhibiting apoptosis and differentiation	[190]
LINC01503	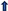		protection of GLI2 from FBXW1-mediated degradation	[191]
HOTAIR	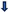	PDCD4	decreases recruitment of EZH2 and LSD1 upregulation of the tumor suppressor PDCD4 inhibits GSC proliferation, invasion, and tumorigenicity	[212]
H19	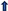		Enhances neurosphere formation Promotes GSC self-renewal and stemness	[213]
MALAT1	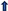	SOX-2, Nestin	Maintains stemness Enhances GSC viability, proliferation, and glioma tumorigenesis	[192,193]
NEAT1	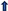	CDK-6	Promotes GSC proliferation, migration, and invasion	[202]
TALNEC2	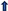		Promoting mesenchymal transformation and self-renewal	[214]
SOX2OT	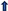		Supports GSC growth and invasion	[215]
CRNDE	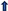		Enhances GSC proliferation, migration, and malignancy Inhibiting miR-384 and miR-186	[216]
circKPNB1	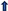	SPI1	Increase GSC viability, proliferation, invasion, neurosphere formation, and stemness	[200]
circRPPH1	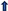		Maintain GSC stemness	[204]
circASPM	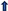	Sponging miR-130b-3p	Promotes GSC proliferation and tumorigenesis	[195]
cARF1	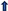	Sponging miR-342-3p	Promotes angiogenesis and tumor progression	[197]
circ-E-Cad	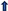	E-cadherin	Promotes GSC maintenance and tumorigenicity	[194]

## 6. Integrated Strategies to Target GSC Survival Circuits

**Figure 5:**
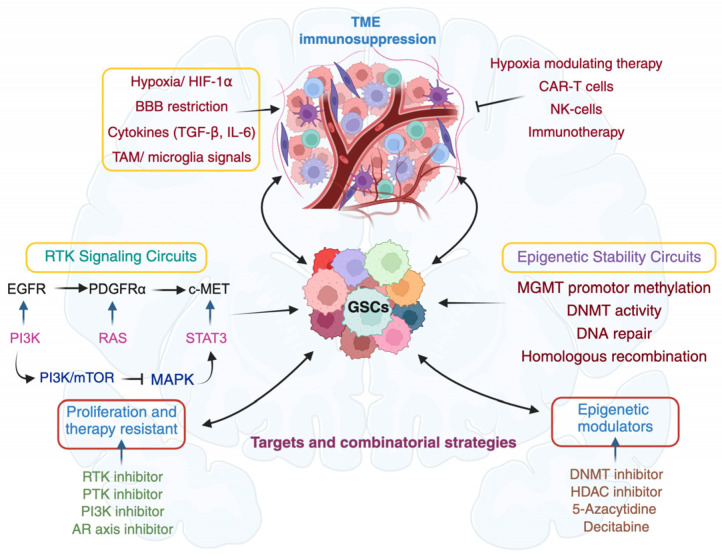
Integrated signaling, epigenetic, and microenvironmental mechanisms sustaining glioma stem cells and therapeutic resistance in glioblastoma. The figure highlights how GSC eradication may require integrated targeting of RTK signaling, epigenetic programs, and the immunosuppressive microenvironment to overcome intrinsic and adaptive resistance mechanisms in glioblastoma. The schematic illustrates the core survival networks that enable glioma stem cells (GSCs) to persist under therapeutic pressure and highlights potential intervention points for combinatorial treatment strategies. Central GSC survival is sustained by three interconnected circuits: (1) receptor tyrosine kinase (RTK) signaling, driven by EGFR, PDGFRα, and c-MET activation, which converge on PI3K/AKT/mTOR, RAS/MAPK, and STAT3 pathways to promote proliferation, metabolic adaptation, and therapy resistance; (2) epigenetic stability, including MGMT promoter methylation, DNMT activity, and chromatin remodeling programs that preserve stemness and reinforce resistance to alkylating agents and radiation; and (3) microenvironmental support, characterized by hypoxia/HIF-1α signaling, limited drug penetration across the blood–brain barrier, cytokine-mediated immunosuppression (TGF-β, IL-6), and tumor-associated macrophage/microglia-derived cues that maintain a protective niche. Therapeutic approaches targeting these circuits aimed at disrupting niche-driven survival signals.

Despite significant advances in neuro-oncology, GBM remains one of the most treatment-refractory human malignancies. Standard-of-care therapy, maximal resection followed by radiotherapy and TMZ, provides only modest benefit, and durable remission is extremely rare. Highly infiltrative growth, rapid adaptive resistance, and the restrictive blood–brain barrier (BBB) collectively limit therapeutic efficacy (Figure 5). Although anti-angiogenic agents and TMZ may prolong progression-free survival in selected patients, overall survival has improved minimally in the past decade [217]. These shortcomings underscore the urgent need for strategies that target the biological engines of recurrence. In this context, GSCs are the central drivers of tumor propagation, therapeutic resistance, and recurrence represent a critical therapeutic focus [218,219].

Receptor tyrosine kinases (RTKs) act as major regulatory hubs integrating extracellular signals into PI3K/AKT/mTOR, RAS/MAPK, and JAK/STAT pathways that sustain GSC growth, invasion, and treatment resistance [220,221]. Genetic amplification, mutation, and ligand dysregulation drive persistent RTK activation. Yet clinical outcomes with RTK-directed therapies have been disappointing [222,223]. Multikinase inhibitors (cabozantinib, dasatinib, pazopanib, pexidartinib) and selective RTK inhibitors demonstrate limited survival benefit, largely due to pathway redundancy, intratumoral heterogeneity, compensatory signaling, and microenvironment-driven resistance [224,225,226,227,228,229,230,231,232,233,234].

EGFR, altered in ~60% of GBM, illustrates these challenges. First-generation inhibitors (gefitinib, erlotinib) and antibody drug conjugates such as depatuxizumab mafodotin have not yielded consistent benefit [235,236,237]. Even next-generation inhibitors (dacomitinib, osimertinib) show limited activity due to robust pathway rewiring and GSC plasticity [238,239,240,241,242,243,244,245,246,247,248]. Combination strategies co-targeting intersecting pathways such as EGFR and androgen receptor signaling may enhance tumor cell death and suppress GSC maintenance [249,250]. Alterations in fibroblast growth factor receptors (e.g., FGFR3-TACC3 fusions) represent actionable nodes in molecularly defined subsets, where erdafitinib and pemigatinib demonstrate early promise [251,252,253,254,255,256]. c-MET signaling similarly contributes to GSC invasion and survival; while capmatinib shows modest activity, crizotinib exhibits improved outcomes in combination settings and synergizes with EGFR blockade [257,258,259,260].

The PI3K/AKT/mTOR axis, one of the most frequently altered pathways in GBM, integrates mitogenic, metabolic, and survival cues [261,262,263]. Multiple PI3K/mTOR inhibitors, dual inhibitors, isoform-selective agents, and ATP-competitive compounds are in clinical evaluation [15,264,265,266]. Nonetheless, many first-generation agents (e.g., dactolisib, voxtalisib) have produced limited benefit due to incomplete pathway suppression or toxicity [267,268,269,270,271,272,273]. Newer ATP-competitive inhibitors that target both mTORC1 and mTORC2 remain in development and may offer improved pathway blockade [274,275]. Collectively, these findings highlight the need for molecular stratification and rational combinations rather than single-agent inhibition. Epigenetic dysregulation further shapes therapeutic response. MGMT promoter methylation remains an important predictive biomarker. DNMT inhibitors (decitabine, 5-azacytidine) modulate gene expression, promote differentiation, and enhance immune recognition that GSCs display, supporting their evaluation as adjunctive agents in ongoing trials (NCT05268666; [133,276,277,278,279,280,281,282,283,284,285,286]).

Immunotherapeutic strategies aim to eliminate GSCs while restoring antitumor immunity. Vaccines, immune checkpoint inhibitors, chimeric antigen receptor T cell therapy (CAR-T) cells, and natural killer (NK) cell-based therapies show promising preclinical activity [287,288,289]. CAR-T cells targeting EGFRvIII or IL13Rα2 selectively eliminate GSCs but are hindered by the profoundly immunosuppressive GBM microenvironment [290,291]. Engineering TGF-β-resistant CAR constructs enhances cytotoxicity and reshapes the immune milieu [292,293]. NK cells, including CAR-NK derivatives and NK-derived exosomes, exhibit potent activity, yet their efficacy is attenuated by GSC-driven immune suppression, including human leukocyte antigen-G (HLA-G) upregulation and inhibitory KIR/HLA interactions [294,295,296].

Although these therapies frequently suppress proliferation and reduce viability in preclinical models, translation into durable clinical benefit remains limited. Multiple biological and pharmacologic barriers contribute to this gap: (1) restricted drug delivery across the BBB and heterogeneous blood–tumor barrier results in inadequate intratumoral drug exposure; (2) intratumoral heterogeneity allows resistant GSC subpopulations to survive despite target inhibition; (3) pathway redundancy and adaptive reprogramming enable rapid compensation even when initial inhibition is effective; and (4) microenvironmental constraints, including hypoxia, immune suppression, and metabolic plasticity, reinforce treatment resistance. Collectively, these challenges explain why single-agent targeted therapies rarely succeed and highlight the necessity of biomarker-guided patient selection, advanced drug-delivery systems, and rational combination therapies that simultaneously address GSC-driven heterogeneity, microenvironmental protection, and adaptive resistance.

## 7. Strategic Advances and Clinical Outlook

Glioblastoma remains one of the most biologically complex and treatment-refractory human cancers. Its lethality is driven not only by extensive genetic and molecular heterogeneity but also by the presence of phenotypically plastic GSCs that shift dynamically across cellular states in response to microenvironmental cues [294]. This adaptability fuels tumor progression, immune evasion, and resistance to standard therapies, severely limiting the efficacy of single-pathway or single-population targeting approaches [295]. Effective therapeutic innovation will therefore require strategies capable of addressing the full spectrum of GSC diversity, disrupting state transitions, and dismantling microenvironmental support systems that maintain tumor plasticity [296,297]. A deeper molecular understanding of GSC-driven heterogeneity is central to advancing precision therapies and improving clinical outcomes in glioblastoma.

### 7.1. Challenges in Targeting GSC Adaptive Plasticity

#### 7.1.1. Molecular and Cellular Heterogeneity

GBM heterogeneity arises through the accumulation of driver and passenger mutations, creating diverse clonal lineages with distinct molecular features [298,299,300]. Although bulk transcriptomic classification into classical, proneural, and mesenchymal subtypes captures broad patterns, single-cell analyses reveal a far more dynamic landscape, with cells traversing NPC-like, OPC-like, AC-like, and MES-like states [301,302,303]. This fluidity underlies treatment resistance, disease progression, and inter-patient variability, highlighting the need for therapeutic strategies that simultaneously account for genetic diversity and state-specific phenotypes.

#### 7.1.2. Plasticity and Stemness Dynamics

GSC identification is complicated by the absence of a universal marker and the ability of stem-like cells to transition across phenotypic states in response to environmental cues, therapeutic pressure, transcriptional reprogramming, and epigenetic remodeling [71,73,304,305]. GSCs occupy a continuum of neurodevelopmental and injury-response states, ranging from stem-like to progenitor-like and differentiated-like phenotypes. Integrative tools such as single-cell profiling, molecular barcoding, and lineage modeling have mapped these trajectories and revealed the transcriptional regulators governing state transitions [22,306,307,308,309]. Transcription factors, including achaete-scute homolog 1 (ASCL1), hairy and enhancer of split 1 (HES1), OLIG2, SOX2, and Notch ligands, mediate quiescence, proliferation, and differentiation, while migratory phenotypes of bipolar glioma cells reflect additional layers of adaptive behavior [310,311,312,313,314]. Therapeutic modulation of these dynamic transcriptional programs may offer a more effective approach than targeting static surface markers.

#### 7.1.3. Epigenetic and Metabolic Adaptability

Dynamic epigenetic remodeling, including DNA methylation, demethylation, and chromatin reorganization, enables GSCs to reconfigure lineage programs and transition between multipotent and more differentiated-like states [315,316,317,318]. This plasticity sustains tumor heterogeneity and promotes resistance but also exposes epigenetic vulnerabilities that may be harnessed therapeutically. GSCs also exhibit remarkable metabolic adaptability. They engage in glycolysis, glutaminolysis, fatty acid oxidation, and pyrimidine synthesis depending on environmental stressors. Hypoxia-driven glycolysis leads to lactate accumulation and acidification, suppressing cytotoxic T cell and NK cell activity while promoting Treg and TAM recruitment. Conversely, TAMs reinforce glycolysis in GSCs, establishing a metabolic–immune feedback loop that stabilizes immune evasion [319,320,321,322,323]. Therapeutic strategies targeting key metabolic pathways (e.g., glycolytic enzymes, GLUT1, or lactate export) may disrupt this circuitry and recondition the TME [324].

#### 7.1.4. Dormancy and Resistance

Necrosis-associated quiescence provides a critical survival mechanism for GSCs located in hypoxic, nutrient-deprived regions. Dormant cells evade therapies that target proliferating populations, later re-entering the cell cycle to regenerate aggressive clones and drive recurrence [183,325,326,327,328,329]. FOXG1, Wnt/β-catenin, and other signaling networks govern dormancy entry and exit, offering potential points of intervention. Therapeutic strategies under investigation include forced reactivation (sensitizing dormant cells to cytotoxic therapies), enforcement of stable dormancy, and niche disruption via CXCR4 antagonists such as plerixafor [26,330,331,332,333]. Beyond dormancy, GSC-mediated chemo- and radioresistance is reinforced by elevated MGMT expression, ABC transporter activity, robust DNA damage response pathways, and upregulation of stemness-related Notch and SHH signaling [145,159,334,335,336].

### 7.2. Future Prospects and Strategies to Overcome Challenges

#### 7.2.1. Integrated Multi-Target Therapies

**Figure 6:**
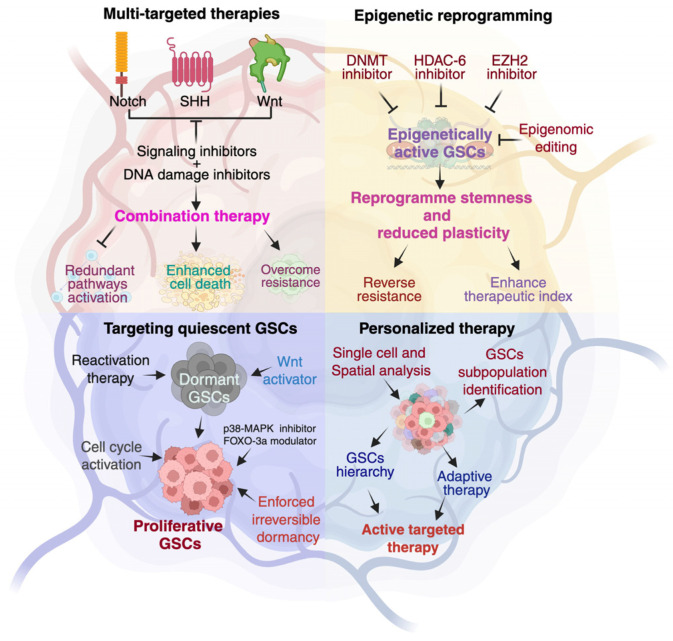
Therapeutic targeting of glioma stem cell plasticity and heterogeneity. Conceptual framework illustrating approaches to overcome glioblastoma recurrence by targeting glioma stem-like cell (GSC) plasticity and hierarchy. (**Top left**) Developmental signaling pathways, including Notch, Sonic Hedgehog (SHH), and Wnt, promote resistance and survival; their inhibition, combined with DNA-damage-based therapies, is proposed to enhance tumor cell death and limit compensatory pathway activation. (**Top right**) Epigenetic reprogramming strategies using DNMT, HDAC6, and EZH2 inhibitors, as well as epigenomic editing, aim to reduce stemness, restrict cellular plasticity, and reverse therapeutic resistance. (**Bottom left**) Quiescent/dormant GSCs evade treatment but can either be forced into proliferation for sensitization or maintained in irreversible dormancy through regulators such as p38-MAPK and FOXO3a modulators. (**Bottom right**) Precision medicine approaches integrate single-cell and spatial analyses to identify GSC subpopulations, define lineage hierarchies, and guide adaptive, active targeted therapies. Together, these strategies address tumor heterogeneity and the dynamic state transitions underlying GBM recurrence.

GSCs survive through tightly interconnected developmental pathways (Notch, SHH, Wnt), oncogenic signaling networks (EGFR, PI3K/AKT/mTOR), and adaptive stress responses. Inhibiting a single node often triggers compensatory activation of parallel pathways, rendering monotherapies ineffective. Multimodal approaches, such as combining SHH or Notch inhibitors with PI3K or HDAC modulators, can suppress redundant signaling and promote synthetic lethality. Integrating pathway inhibitors with DNA-damaging agents (e.g., TMZ, PARP inhibitors) may further exploit vulnerabilities in DNA repair capacity. Such rational combinations address intratumoral heterogeneity and align therapy with patient-specific molecular profiles (Figure 6). Alpha-1,3-mannosyl-glycoprotein 4-beta-N-acetylglucosaminyltransferase A (MGAT4A) drives glioblastoma stem cell tumorigenicity by N-glycosylating EGFR at N604, activating EGFR-ERK1/2 signaling and promoting self-renewal, proliferation, and invasion [337]. Genetic, pharmacologic, and CRISPR-nanotherapeutic targeting suppresses invasion and prolongs survival in orthotopic models, identifying glycosylation-dependent EGFR signaling as a clinically actionable vulnerability in GBM.

#### 7.2.2. Epigenetic Reprogramming to Limit Plasticity

Epigenetic regulators such as EZH2, KDM4A, DNMT3A, and HDACs maintain chromatin states that confer GSC stemness and resistance. Pharmacologic inhibition of these enzymes (e.g., tazemetostat, decitabine, vorinostat) can restore tumor-suppressive gene expression, enforce differentiation, and enhance sensitivity to radiotherapy or chemotherapy (Figure 6). CRISPR-based epigenome editing platforms offer emerging opportunities for precise and reversible modulation of regulatory loci, selectively reprogramming malignant epigenetic states while sparing normal neural stem cells.

#### 7.2.3. Targeting Dormancy and Quiescence

Dormant GSCs persist as a reservoir for recurrence. Strategies aim either to reactivate dormant cells, rendering them vulnerable to cytotoxic therapy, or to induce irreversible quiescence. Modulation of BMP, Wnt/β-catenin, p38 MAPK, ERK1/2, and IGF-1R pathways shows promise in shifting dormant populations from resistant to therapy-susceptible states (Figure 6). Niche-targeting strategies, such as CXCR4 inhibition, may mobilize dormant GSCs and enhance drug exposure.

#### 7.2.4. Precision Oncology and Single-Cell-Guided Therapeutics

Traditional bulk profiling obscures the cellular diversity underlying GBM resistance. Single-cell and spatial multi-omics now enable high-resolution mapping of GSC subpopulations and microenvironmental interactions. Integration of transcriptomic, epigenomic, and proteomic datasets allows identification of patient-specific vulnerabilities and informs adaptive, real-time therapeutic design. Patient-derived organoids and xenografts serve as functional platforms for validating individualized combinations. Fluorogenic tetrazine probes enable real-time imaging of GSC-associated receptors, metabolism, and niche interactions in vivo. Coupled with click-to-release or pretargeting strategies, they allow localized drug activation, bridging molecular GSC insights with precision imaging and targeted therapy [338].

#### 7.2.5. Microenvironmental Disruption

**Figure 7:**
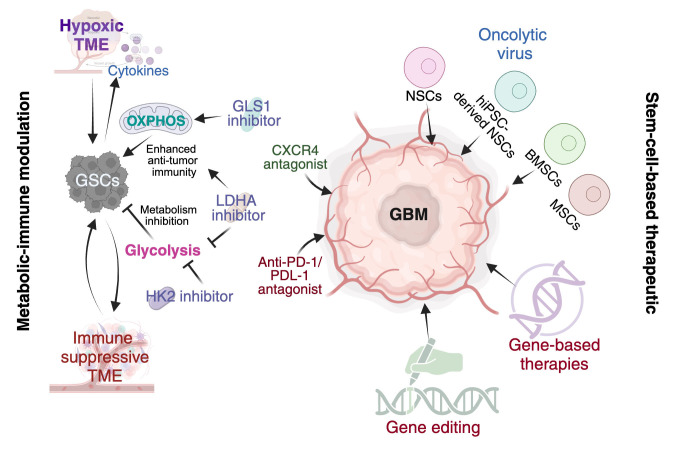
Metabolic–immune crosstalk and emerging therapeutic interventions in glioblastoma. Schematic overview of how the glioblastoma (GBM) microenvironment sustains tumor growth and therapy resistance, and how current and emerging treatments aim to disrupt these processes. A hypoxic tumor microenvironment (TME) promotes cytokine production and metabolic reprogramming, enhancing oxidative phosphorylation (OXPHOS) and glycolysis in glioma stem-like cells (GSCs). These metabolic states support stemness and contribute to the formation of an immunosuppressive niche. Targeting tumor metabolism with GLS1, LDHA, and HK2 inhibitors is proposed to impair GSC maintenance and viability. In parallel, immune evasion mechanisms are addressed through immune checkpoint blockade (anti-PD-1/PD-L1) and CXCR4 antagonism to improve immune infiltration. Additional experimental approaches include gene-based therapies, gene editing, and oncolytic viruses designed to selectively infect tumor cells and stimulate anti-tumor immunity. Collectively, the figure highlights coordinated metabolic and immune dependencies in GBM and therapeutic strategies aimed at disrupting these interconnected resistance pathways.

The GSC niche, particularly perivascular and hypoxic regions, provides structural support, metabolic resources, and immune protection. Therapeutic disruption of these niches through CXCL12-CXCR4 blockade, vascular endothelial growth factor (VEGF)/angiopoietin-2 (ANGPT2) inhibition, or extracellular matrix modulation can deprive GSCs of essential cues and improve treatment penetration (Figure 7). Combining microenvironment-disrupting agents with anti-angiogenic therapy may prevent niche reconstitution and limit invasion.

#### 7.2.6. Stem-Cell-Based Therapeutic Platforms

Stem cells, including NSCs, MSCs, and iPSC-derived NSCs, possess intrinsic tumor-homing properties that enable delivery of therapeutic payloads to infiltrative and hypoxic tumor regions inaccessible to conventional drugs [339,340,341,342,343,344,345,346,347]. Engineered stem cells expressing suicide genes, oncolytic viruses, TGF-β inhibitors, or replicating adenoviruses facilitate localized cytotoxicity (Figure 7) while minimizing systemic toxicity [348,349,350,351,352,353,354]. iPSC-derived platforms offer additional advantages, including patient-specific compatibility and ease of genetic manipulation [355,356].

#### 7.2.7. Metabolic–Immune Axis Modulation

GSC metabolic flexibility sustains tumor growth and shapes immune dysfunction. Targeting glycolytic enzymes (HK2, LDHA), glutaminolysis (GLS1), or mitochondrial respiration (CPI-613, metformin) can impair survival and sensitize GSCs to treatment. Inhibiting lactate export (MCT1/4) may restore T cell function and synergize with immune checkpoint blockade or CXCR4 antagonists. Such approaches are particularly relevant in recurrent GBM, where metabolic adaptation intensifies treatment resistance (Figure 7).

Collectively, these approaches represent a paradigm shift toward integrative, precision-guided therapeutics designed to confront the heterogeneity, plasticity, and niche dependence of GSCs. Convergent strategies incorporating multi-pathway inhibition, epigenetic reprogramming, metabolic targeting, microenvironmental disruption, and stem-cell-guided delivery aim to achieve deeper, more durable clinical responses. Integration of patient-specific modeling systems will further enable rational deployment of these interventions, aligning treatment selection with the molecular and cellular landscape of each tumor. Such advancements hold strong potential for sustained disease control and improved outcomes in glioblastoma.

## 8. Conclusions

GSCs are the central architects of glioblastoma lethality, driving tumor initiation, plasticity, therapy resistance, and immune escape. Their dynamic transitions, metabolic flexibility, and dependence on specialized niches create a self-sustaining network that enables recurrent growth despite aggressive multimodal therapy. This review highlights how genetic diversity, epigenetic remodeling, metabolic rewiring, and non-coding RNA circuits converge to preserve GSC identity and reinforce resistance to conventional single-pathway inhibition. These insights signal a necessary therapeutic shift toward integrative, precision-based strategies that simultaneously target GSC-intrinsic programs and the microenvironmental cues supporting their plasticity. Promising avenues include multimodal pathway inhibition to counteract signaling redundancy, epigenetic reprogramming to restrict stemness, metabolic–immune axis modulation to restore antitumor immunity, and stem cell-based delivery systems to reach infiltrative and treatment-shielded tumor regions. Combinatorial approaches targeting interconnected signaling pathways, epigenetic vulnerabilities, metabolic–immune adaptations, and leveraging stem cell-based delivery platforms offer the most compelling route to durable control. Coupled with single-cell and spatial profiling to guide patient-specific therapy design, these emerging strategies outline a realistic path toward overcoming resistance and improving outcomes in glioblastoma.

## Data Availability

No new data were created or analyzed in this study.

## Abbreviations

The following abbreviations are used in this manuscript:

ABC	ATP-binding cassette
ANGPT2	Angiopoietin-2
AKT	Protein kinase B
ALDH1A1/3	Aldehyde dehydrogenase 1A1/1A3
AR	Androgen receptor
ATM	Ataxia telangiectasia mutated
ATR	Ataxia telangiectasia and Rad3-related
BBB	Blood–brain barrier
BMI1	B lymphoma Mo-MLV insertion region 1
BMP	Bone morphogenetic protein
BRCA1	Breast cancer gene 1
CAR-T	Chimeric antigen receptor T cell
CD	Cluster of differentiation
CHK1/2	Checkpoint kinase 1/2
circRNA	Circular RNA
CNS	Central nervous system
CSC	Cancer stem cell
CXCL12	C-X-C motif chemokine ligand 12
CXCR4	C-X-C motif chemokine receptor 4
DDR	DNA damage response
EGFR	Epidermal growth factor receptor
EGFRvIII	Mutant epidermal growth factor receptor variant III
EMT	Epithelial–mesenchymal transition
EV	Extracellular vesicle
FGFR	Fibroblast growth factor receptor
FIH1	Factor-inhibiting hypoxia-inducible factor 1
GBM	Glioblastoma
GLI	Glioma-associated oncogene homolog
GSC	Glioblastoma stem cell
H2AX	H2A histone family member X
H3K9me3	Histone H3 lysine 9 trimethylation
HGF	Hepatocyte growth factor
HGFR (c-MET)	Hepatocyte growth factor receptor
HIF-1α	Hypoxia-inducible factor 1 alpha
IDH1	Isocitrate dehydrogenase 1
IL	Interleukin
ITGA2	Integrin alpha 2
JAK/STAT	Janus kinase/signal transducer and activator of transcription
lncRNA	Long non-coding RNA
MAPK	Mitogen-activated protein kinase
MES	Mesenchymal
MGMT	O6-methylguanine-DNA methyltransferase
MIC-1	Macrophage inhibitory cytokine-1
miRNA (miR)	MicroRNA
MDSC	Myeloid-derived suppressor cell
MHC	Major histocompatibility complex
mTOR	Mechanistic target of rapamycin
NF-κB	Nuclear factor kappa B
NK cell	Natural killer cell
Notch	Notch signaling pathway
OXPHOS	Oxidative phosphorylation
PD-1/PD-L1	Programmed cell death protein 1/ligand 1
PGE2	Prostaglandin E2
PI3K	Phosphoinositide 3-kinase
PN	Proneural
PPP	Pentose phosphate pathway
PrPC	Cellular prion protein
qGSC	Quiescent glioblastoma stem cell
pGSC	Proliferative glioblastoma stem cell
RTK	Receptor tyrosine kinase
SHH	Sonic Hedgehog
snoRNP	Small nucleolar ribonucleoprotein
STAT3	Signal transducer and activator of transcription 3
TAM	Tumor-associated macrophage
TGF-β	Transforming growth factor beta
TIMP-2	Tissue inhibitor of metalloproteinases-2
TMZ	Temozolomide
TME	Tumor microenvironment
VEGF	Vascular endothelial growth factor
Wnt	Wingless-related integration site
